# Synthesis of Polyhydroxybutyrate Particles with Micro-to-Nanosized Structures and Application as Protective Coating for Packaging Papers

**DOI:** 10.3390/nano7010005

**Published:** 2016-12-30

**Authors:** Vibhore Kumar Rastogi, Pieter Samyn

**Affiliations:** Freiburg Institute for Advanced Studies (FRIAS), Chair for Bio-Based Materials Engineering, Faculty for Environment and Natural Resources, University of Freiburg, Werthmannstrasse 6, 79085 Freiburg, Germany; Pieter.Samyn@outlook.be

**Keywords:** polyhydroxybutyrate, nanofibrillated cellulose, paper coating, hydrophobicity

## Abstract

This study reports on the development of bio-based hydrophobic coatings for packaging papers through deposition of polyhydroxybutyrate (PHB) particles in combination with nanofibrillated cellulose (NFC) and plant wax. In the first approach, PHB particles in the micrometer range (PHB-MP) were prepared through a phase-separation technique providing internally-nanosized structures. The particles were transferred as a coating by dip-coating filter papers in the particle suspension, followed by sizing with a carnauba wax solution. This approach allowed partial to almost full surface coverage of PHB-MP over the paper surface, resulting in static water contact angles of 105°–122° and 129°–144° after additional wax coating. In the second approach, PHB particles with submicron sizes (PHB-SP) were synthesized by an oil-in-water emulsion (o/w) solvent evaporation method and mixed in aqueous suspensions with 0–7 wt % NFC. After dip-coating filter papers in PHB-SP/NFC suspensions and sizing with a carnauba wax solution, static water contact angles of 112°–152° were obtained. The intrinsic properties of the particles were analyzed by scanning electron microscopy, thermal analysis and infrared spectroscopy, indicating higher crystallinity for PHB-SP than PHB-MP. The chemical interactions between the more amorphous PHB-MP particles and paper fibers were identified as an esterification reaction, while the morphology of the NFC fibrillar network was playing a key role as the binding agent in the retention of more crystalline PHB-SP at the paper surface, hence contributing to higher hydrophobicity.

## 1. Introduction

Inspired by nature, surface science is continuously advancing towards deeper analysis and understanding of natural materials in order to further exploit them in modern technology. The exceptional properties and functionality of materials in nature often rely on their hierarchical structure combining features at the micro- to nano-length scales. As such, the development of biomimetic materials is continuously growing by modeling biological surfaces, such as lotus leafs, pitcher plant or insect wings, for the fabrication of artificial superhydrophobic surfaces [1,2,3,4].

Surface hydrophobicity is a primary requirement for the creation of a protective barrier layer on papers and can be adjusted by tuning both chemical surface components and surface morphology. Nowadays, the hydrophobicity of a paper product is still traditionally enhanced by internal sizing and/or surface sizing. The internal sizing process involves the addition of cellulose reactive chemicals, such as rosin-alum, alkenyl succinic anhydrate (ASA) and alkyl ketene dimer (AKD), to the pulp stock solution [5]. The required hydrophobicity can then be achieved only after the paper drying. In the case of surface sizing, water-resistant polymers, such as petroleum-based polyolefins (e.g., polyethylene), polyvinylidene chloride (PVDC) and waxes, or bio-based polymers, such as polylactic acid (PLA) [6] and polyhydroxybutyrate (PHB) [7], are coated directly over the dried paper web or in combination with binders, like styrene butadiene latex and styrene acrylic latex. Several coating techniques are available in the industry for the application of polymers over papers, such as extrusion, dispersion coating and solvent coating [8]. However, thermoplastic polymers, such as PHB, have indicated difficulties in processing via extrusion due to the melt instability near melting temperatures [9]. Therefore, PHB has been coated over paper by solvent casting from chloroform, showing water contact angles in the range of 95°–80° for PHB concentrations of 2–10 *w*/*v* % [10]. However, such treatment will not lead to further improvement in the water contact angle (>100°), due to the flat surface morphology and intrinsic hydrophobicity of PHB. In addition, surface hydrophobicity of paper can be further enhanced by increasing the roughness of the existing hydrophobic surface through the creation of a surface profile with multiple length scales. As such, hierarchical micro- to nano-scale surface patterns can be generated by following mechanical stretching and/or etching techniques, such as plasma, laser and chemical etching [11], lithography [12], the sol-gel process [13], electrochemical deposition, layer-by-layer and colloidal assembly [14]. Similarly, the surface hydrophobicity of papers can be enhanced by controlling the surface morphology through deposition of micro- to nano-scale structured particles, providing hierarchical structures in combination with the macroscale fibers.

Several inorganic nanoparticles, such as TiO_2_, SiO_2_ and Al_2_O_3_, coupled with low surface energy chemicals, like 3-(trimethoxysilyl) propyl methacrylate (MPS) or silanes, acted as highly hydrophobic materials when coated over papers [15,16]. In addition, a paper coating with organic nanoparticles of styrene maleimide showed high surface hydrophobicity and improved water repellency with self-cleaning ability [17,18]. However, only a few articles described the fabrication of highly hydrophobic papers obtained by purely using biopolymers as a coating. In one study, cellulose has been exploited as a nanoscale coating for the fabrication of superhydrophobic papers using porous structured microparticles of surface-modified nanofibrillated cellulose (NFC) [19]. The microparticles were formed by spray drying of solvent-based NFC, followed by quick drying and modification by fluorinated trichlorosilane surfactant. Similarly, cellulose stearoyl ester nanoparticles were made from solution via nanoprecipitation and spray-coated over papers to provide structured superhydrophobic surfaces with a water contact angle of more than 150°, exhibiting self-cleaning properties [20], while the surface hydrophobicity of these papers can be further tuned by thermal annealing. In another example, the PHB polymer was applied on paper and transformed into micro- or nano-particles through a phase-separation technique in direct contact with paper, providing water contact angles of 153° [21]: however, the need for long immersion times (12 h) of the native PHB-coated paper in an ethanol/water coagulation bath may result in the swelling of paper fibers and a decrease in the mechanical properties. The application of PHB as a food packaging material has also been critically described in a review, indicating the advantages, drawbacks and several routes for overcoming these drawbacks [22]. Advantageously, the integration of NFC may further improve the barrier properties due to the intrinsic oxygen barrier resistance of fibrillated cellulose, as demonstrated before in combinations with PLA [23] and shellac [24]. However, the hydrophobic protection of the hydrophilic NFC is a primary requirement to maintain barrier resistance properties in a complex coating system.

In this work, two approaches for improving the hydrophobicity of packaging papers through the creation of hierarchical structures on the paper surface will be presented, using fully-bio-based additives, including polyhydroxybutyrate (PHB) and nanocellulose (NFC). In the first approach, internally-structured micro-sized polyhydroxybutyrate particles (PHB-MP) will be prepared. In the second approach, aqueous suspensions containing non-structured submicron-sized particles (PHB-SP) mixed with nanofibrillated cellulose (NFC) as a binding agent will be prepared. Finally, the particles will be transferred as a coating onto paper surfaces by a facile two-step dip-coating process into: (i) a suspension of PHB-MP or PHB-SP/NFC; followed by (ii) a plant wax solution as the hydrophobizing agent. After synthesis and analytical characterization of the particles, the morphology of coating formulations was evaluated and optimized to achieve the highest hydrophobicity.

## 2. Results and Discussion

### 2.1. Synthesis and Characterization of PHB Microparticles and Submicron Particles

#### 2.1.1. Morphological Analysis by Scanning Electron Microscopy

**Figure 1:**
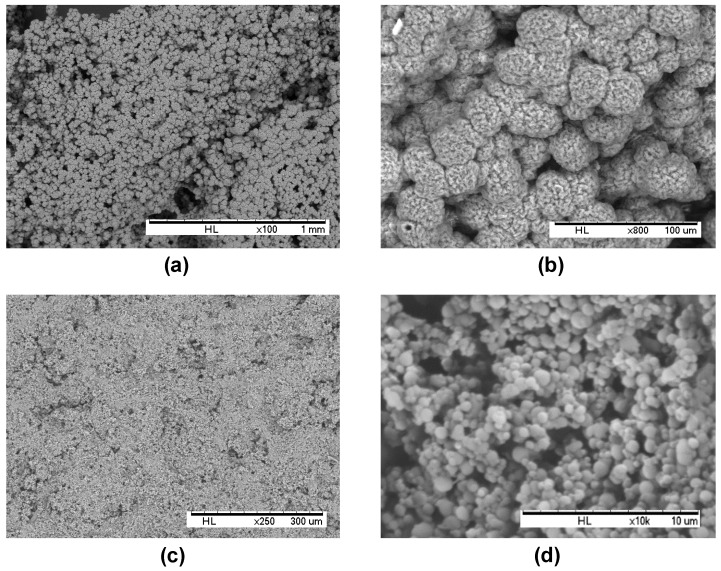
Scanning electron microscopy of (**a**,**b**) internally-structured polyhydroxyalkanoate microparticles (PHB-MP), and (**c**,**d**) non-structured polyhydroxyalkanoate submicron particles (PHB-SP), (note the different scales of magnification for the best representation of the particle morphology).

The morphology of dried PHB-MP and PHB-SP powders was studied by scanning electron microscopy (SEM) (Figure 1). The spherical micro-sized PHB-MP have an average diameter of 25 ± 5 µm and clearly show well-developed internal structures, as visualized in Figure 1a,b (magnification). The PHB-MP were synthesized by following a phase-separation technique [21], with some modifications: dimethylformamide (DMF) was used to dissolve PHB pellets instead of chloroform, with the advantage of dissolving more PHB in smaller amounts of solvent in less time. The formation of PHB-MP can be understood as a result of the exchanges between solvent and non-solvent in the system, where an ethanol/water mixture (acting as a non-solvent for PHB) diffuses into the PHB/DMF solution and induces the precipitation of PHB as spherical micro-sized particles. The exchange between solvent and non-solvent may result in a thermodynamic instability and phase separation of PHB. It is important to note that the spherical PHB-MP developed in the suspension only, just after the addition of the non-solvent; whereas, the creation of internal structures within PHB-MP can be explained as a result of drying PHB/DMF/ethanol-water suspension at 70 °C, which facilitates the fast evaporation of ethanol (boiling point = 78 °C) and the simultaneous slow evaporation of DMF (boiling point = 153 °C). This resulted in self-organization of the PHB molecules at the solvent interfaces, and hence, the formation of nanoscale internal structures within PHB-MP.

The spherical submicron-sized PHB-SP have an average diameter of 800 ± 300 nm and do not show further internal structuring, as illustrated in Figure 1c,d (magnification). The PHB-SP were synthesized by following the oil-in-water emulsion (o/w) solvent evaporation method [25], with slight modifications: the PHB was dissolved in chloroform and acted as an oil phase in the external water phase containing polyvinyl alcohol (PVA), a synthetic polymer that was used as an emulsifier instead of anionic/cationic detergents. A low concentration of PVA (0.1 *w*/*v* %) in the external water phase was chosen to ensure the low viscosity of the o/w emulsion, resulting in a more easy break-up of the emulsion into smaller droplets, which get stabilized to avoid coalescence. Further, the slow rate of solvent evaporation at low pressure (200 mbar) and low temperature (40 °C) causes the slow diffusion of chloroform out of the emulsion droplets and assists in the formation of solid non-structured submicron-sized PHB particles.

#### 2.1.2. Thermal Analysis

In order to understand the effects of synthesis method on the thermal stability and crystallinity of the PHB particles, thermal analysis was done on neat PHB as a reference material, together with dried PHB-MP and PHB-SP powders. The thermal properties were analyzed by thermogravimetric analysis (TGA) and differential scanning calorimetry (DSC), as discussed below.

**Figure 2:**
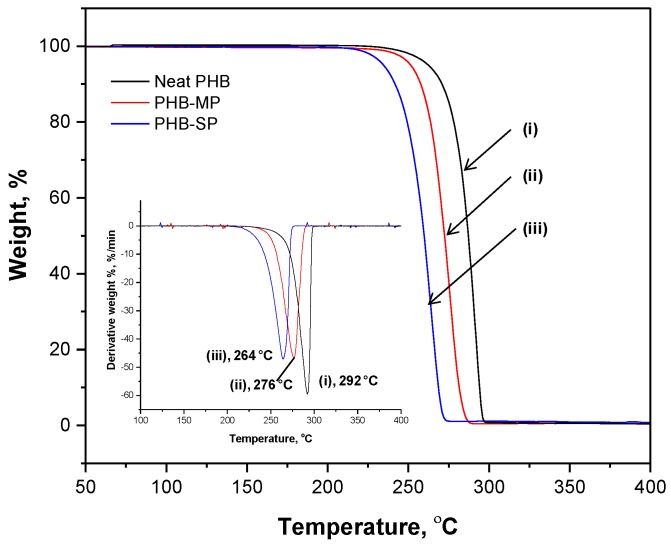
Thermogravimetric analysis (TGA) representing weight loss and differential weight loss (inset) curves of (i) neat PHB, (ii) PHB-MP and (iii) PHB-SP.

The thermal degradation of neat PHB, PHB-MP and PHB-SP at temperatures between 50 °C and 400 °C is illustrated by the weight loss curve according to TGA analysis (nitrogen atmosphere) in Figure 2. The thermal degradation of all materials took place in a single step, representing the narrow processing window of the polymer. At temperatures up to 200 °C, full stability of all materials is observed, with less than 1% of residual adsorbed water and no significant mass loss where most of the trapped solvents would have evaporated. As detailed in a derivative weight loss curve (Figure 2, inset), the neat PHB presents a higher degradation temperature (292 °C) than PHB-MP (276 °C) and PHB-SP (264 °C). The variations in thermal stability can be mainly explained by size effects: the smaller submicron size of PHB-SP, resulting in a higher free surface area, is thermally less stable than the original densely-compacted neat PHB pellets; whereas, the thermal stability of PHB-MP lies intermediately between neat PHB and PHB-SP due to the porous micron size structure, having less free surface area than PHB-SP and more surface area than neat PHB.

**Figure 3:**
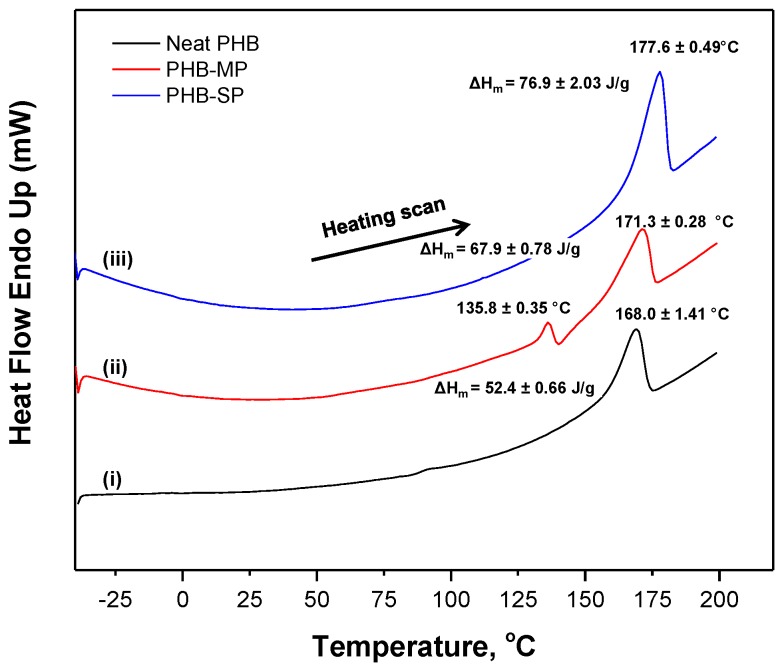
Differential scanning calorimetry (DSC) thermographs of (i) neat PHB, (ii) PHB-MP and (iii) PHB-SP.

The melting behavior of neat PHB, PHB-MP and PHB-SP was analyzed by the thermographs according to DSC analysis (nitrogen atmosphere) in Figure 3. A single heating scan was applied to analyze the quality of crystals formed after synthesis of PHB particles, which would otherwise vanish during the subsequent cooling and second heating scan. A single melting peak (*T_m_*_1_) is observed for both neat PHB and PHB-SP, while two melting peaks (*T_m_*_1_, *T_m_*_2_) are observed for PHB-MP. The behavior with a single melting point is usually due to the melting of crystals with uniform lamellar thickness, while multiple melting peaks can be attributed to crystals with different lamellar thickness having different melting temperatures [26]: this indicates that the processing of PHB-SP has not significantly altered the crystal structures, while the instability caused by solvent exchange during the fabrication of PHB-MP resulted in crystals with different sizes and lamellar thickness exhibiting a multiple melting behavior. The melting temperatures for PHB-MP (*T_m_*_2_ = 171.3 °C) and PHB-SP (*T_m_*_1_ = 177.6 °C) were higher than neat PHB (*T_m_*_1_ = 168.0 °C): this indicates the higher degree of crystallinity because of improved polymer chain mobility through solvent processing and hence better crystallization of PHB. The degree of crystallinity (*Χ_c_*) was determined by Equation (1) [27]:

(1) $$X_{c}=\;\frac{\Delta H_{m}}{\Delta H_{m}^{o}}\times 100$$

where Δ*H_m_* represents the melting enthalpy of the crystals formed in the PHB polymer; $\Delta H_{m}^{o}$ is the theoretical enthalpy value for a 100% crystalline PHB and chosen as 146.6 J/g [27]. As such, *X_c_* = 35.74% for neat PHB, 46.32% for PHB-MP and 52.46% for PHB-SP. The highest crystallinity was achieved for PHB-SP, as only one solvent (chloroform) was used in processing, resulting in a better crystallization process due to improved mobility of the PHB polymer chains allowing for structural rearrangements. Otherwise, the diffusion of non-solvents (ethanol/water) into the PHB-DMF solution during the synthesis of PHB-MP resulted in instability and restricted PHB polymer chain mobility, causing a lower degree of crystallinity. The latter restrictions also explain the occurrence of a double melting peak observed for PHB-MP.

#### 2.1.3. Chemical Analysis by Fourier-Transform Infrared Spectroscopy

**Figure 4:**
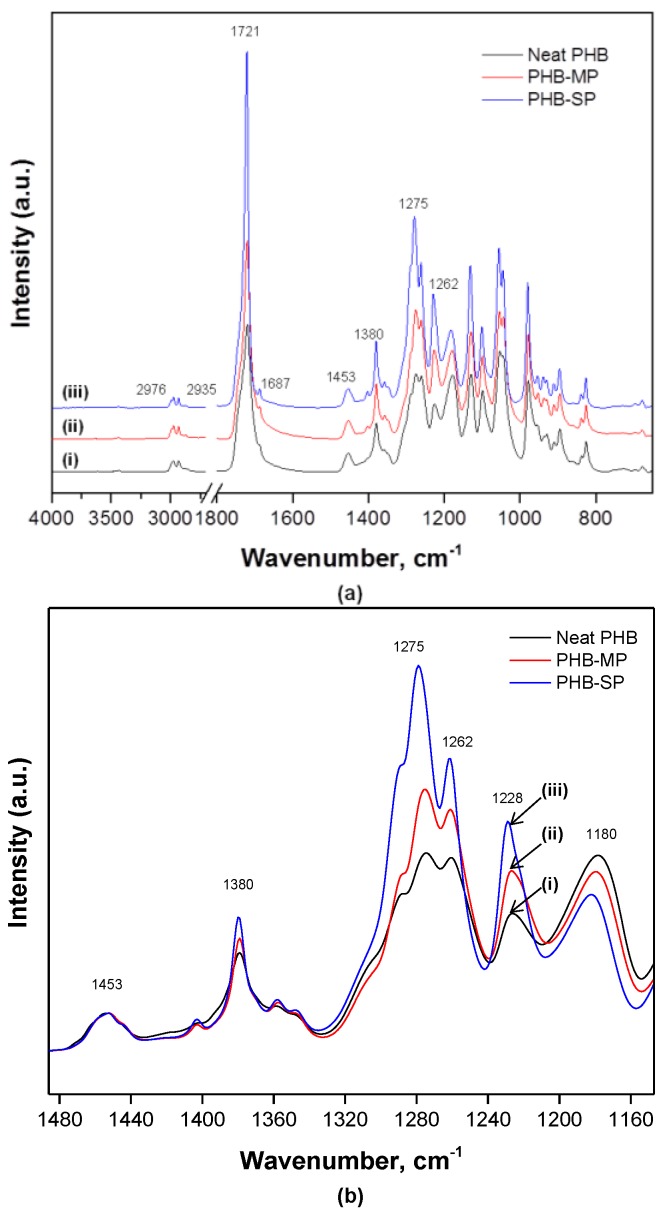
Fourier-transform infrared (FTIR) spectra showing (**a**) complete wavenumber range 4000–400 cm^−1^; (**b**) detailed spectra in wavenumber range 1500–1100 cm^−1^ for (i) neat PHB, (ii) PHB-MP and (iii) PHB-SP.

Both PHB-MP and PHB-SP were characterized by Fourier-transform infrared (FTIR) spectroscopy to evaluate the influences of synthesis methods on the PHB crystallinity, while neat PHB was included as a reference material. The FTIR spectra in Figure 4 show the characteristic absorption bands for PHB, as follows: 2976 cm^−1^ (CH_3_ stretching), 2935 cm^−1^ (CH_2_ stretching), 1721 cm^−1^ (C=O asymmetrical stretching), 1687 cm^−1^ (C=O symmetrical stretching), 1453 cm^−1^ (CH_3_ asymmetric stretching), 1380 cm^−1^ (CH_3_ symmetric stretching), 1275 cm^−1^ (CH_2_ wagging), 1262 cm^−1^ (C–O–C stretching with C–H deformation) and 1228 cm^−1^ (C–CH_3_ stretching) [28,29,30]. The spectra were baseline corrected and normalized on the 1453 cm^−1^ band, which is insensitive for changes in crystallinity [30].

From Figure 4a, the absence of a broad band (3100–3600 cm^−1^) assigned to O–H stretching due to strong intra/inter-molecular hydrogen bonding in PVA confirmed the complete removal of PVA from PHB-SP after washing with water, as PVA is water soluble [31].

From Figure 4b, the wavenumber region 1100–1500 cm^−1^ is detailed for a qualitative analysis of crystallinity for neat PHB, PHB-MP and PHB-SP. The absorption intensity of bands at 1275 cm^−1^ and 1228 cm^−1^ (crystalline bands) and 1180 cm^−1^ (amorphous band) is sensitive to the degree of crystallinity [30]. In semi-crystalline PHB (the present case), all three bands above are present, while in fully-crystalline PHB, only the 1228 cm^−1^ band is present and assigned to the conformation of the PHB helical chains [30]. Considerable differences are observed in the intensity ratio *I*_1228_/*I*_1443_ (crystalline index): the crystallinity index equals 5.7 (PHB-SP), 4.5 (PHB-MP) and 3.4 (neat PHB). The high crystallinity for PHB-SP accounts for better crystallization due to processing with only one solvent. The lower crystallinity for PHB-MP can be ascribed to the instability caused by non-solvents during the synthesis process; whereas, for neat PHB, lowest crystallinity is attributed to the unprocessed state of PHB after its extraction from the microorganisms, which is obviously more amorphous than processed PHB [25]. This trend is in agreement with the previous values for the degree of crystallinity (*X_c_*) calculated from DSC analysis.

### 2.2. First Approach: Application of PHB-MP as Paper Coating

#### 2.2.1. Morphological Analysis (SEM) and Water Contact Angle Measurements

**Figure 5:**
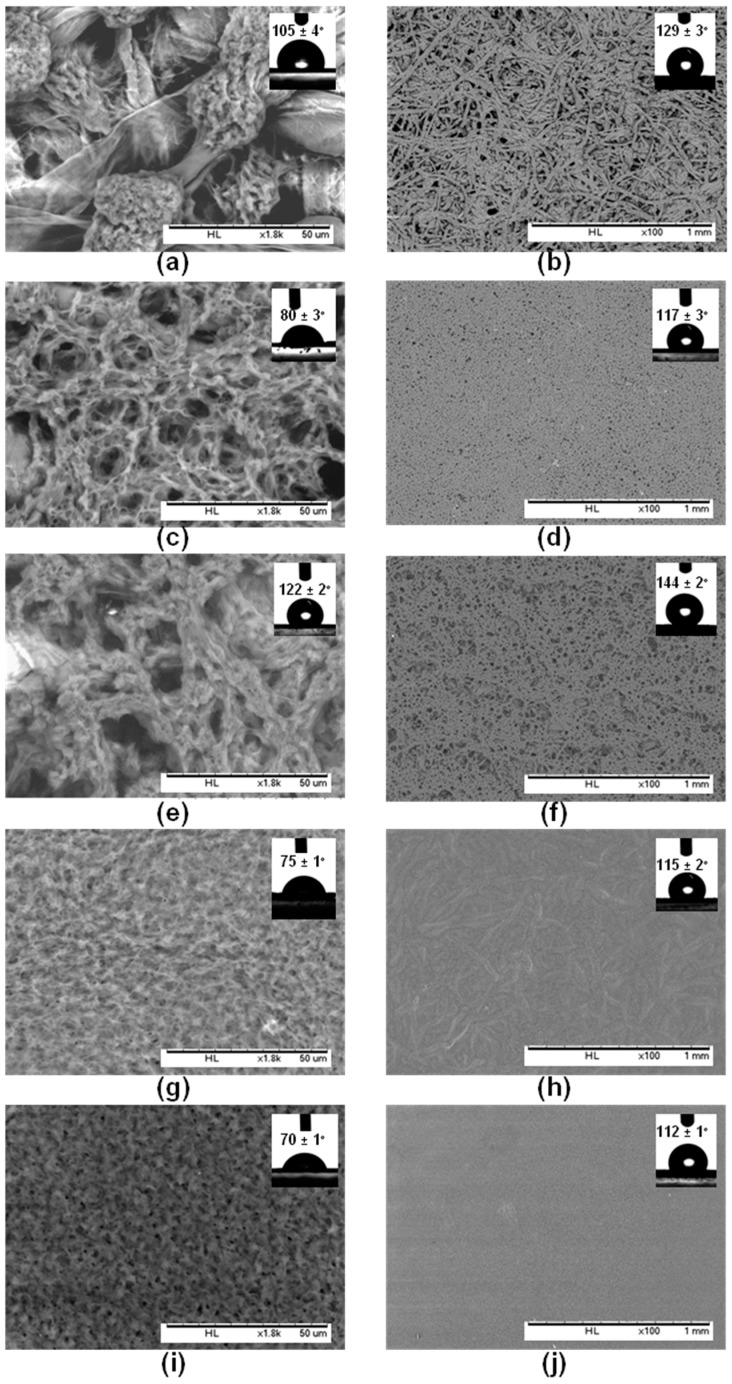
Scanning electron microscopy for PHB-MP-coated papers from suspensions with different concentrations: (**a**,**b**) PHB-MP, with 8.5 *w*/*v* % of PHB and 7.4 *v*/*v* % non-solvent; (**c**,**d**) PHB-MP, with 17.0 *w*/*v* % of PHB and 7.4 *v*/*v* % non-solvent; (**e**,**f**) PHB-MP, with 17.0 *w*/*v* % of PHB and 14.8 *v*/*v* % non-solvent; (**g**,**h**) PHB-MP, with 17.0 *w*/*v* % of PHB and 23.1 *v*/*v* % non-solvent; (**i**,**j**) neat PHB, with 8.5 *w*/*v* % of PHB without non-solvent. The figures (**a**,**c**,**e**,**g**,**i**) are magnified images with a scale of 50 µm; insets are water contact angles on coated papers without additional wax coating. The figures in (**b**,**d**,**f**,**h**,**j**) are images of a larger area with a scale of 1 mm; insets are water contact angles after the application of an additional wax layer over the coated papers.

In the first approach, the PHB-MP were deposited onto paper by dip-coating filter paper samples into the prepared PHB-MP suspension (8.5 *w*/*v* % of PHB and 7.4 *v*/*v* % non-solvent) and drying at 70 °C for 5 h. From a detailed SEM image of the coated papers (Figure 5a), the PHB-MP deposits on paper retain the same size (diameter 25 μm) and internally-nanosized structures, like the PHB-MP powders discussed before. As explained before, the microparticles were formed in suspension after adding the non-solvent, while the nanoscale internal structures formed during drying (here, in contact with paper). However, the PHB-MP on paper were not deposited in close proximity to each other and did not form a compact coating structure, but only a partial surface coverage (Figure 5b). Interestingly, the PHB-MP on paper were mainly localized near the cellulose fibers, suggesting favorable interactions between the particles and cellulose fibers (see further Section 2.2.2). The initial interaction between PHB-MP and paper fibers took place within the wet coating state, when PHB-MP were in amorphous state due to the presence of solvents. In amorphous state, the PHB acts as a sticky material [32] and, hence, chemically adheres to the paper fibers, while these interactions were further strengthened during the drying process, resulting in strong bonds between PHB-MP and paper fibers. Therefore, the preferential interaction of PHB-MP and cellulose fibers may hinder the formation of a continuous coating layer at a given concentration.

The effect of PHB and non-solvent concentrations in suspension on the final (i.e., dry) coating morphology was further investigated. An increase of PHB concentration (up to a maximum of 17 *w*/*v* %) resulted in the formation of no well-defined PHB-MP structures (Figure 5c) and a non-structured layer covering almost the entire paper surface (Figure 5d). This observation can be explained by the insufficient amount of non-solvents (7.4 *v*/*v* %) that would be required for the self-organization of PHB into micro-sized particles. With an increase in the non-solvents’ concentration (up to 14.8 *v*/*v* %), a coating layer with better resolved internal structures developed (Figure 5e). It seems that the PHB-MP formed in the suspension are deposited as random structured moieties over the paper (Figure 5f) rather than clear micro-sized PHB particles, which can be attributed to the favored interactions between PHB and paper fibers at higher PHB and non-solvent concentration. With a high concentration of PHB (17 *w*/*v* %) and non-solvents (14.8 *v*/*v* %), individual cellulose fibers were fully coated due to the increased availability of PHB and equivalent amount of non-solvent available for creating structured particles. The further increase in the non-solvent concentration (up to 23.1 *v*/*v* %) resulted in a flat coating layer without internal structures (Figure 5g), covering the paper surface completely and homogenously (Figure 5h). The very high amounts of non-solvent induced the precipitation of PHB to a maximum extent and resulted in the deposition of a flat layer rather than the formation of microparticles. A similar flat layer was obtained when paper was coated with a PHB suspension (8.5 *w*/*v* % PHB) without non-solvent (Figure 5i,j). Based on this, the concentrations of PHB and non-solvents in suspension were optimized for creating structured PHB-MP over the paper surface, with the possibility for obtaining either single fiber deposits or full surface coverage.

The surface hydrophobicity of PHB-MP-coated papers (8.5–17 *w*/*v* % PHB; 7.4–23.1 *v*/*v* % non-solvents in coating suspensions) was evaluated by static water contact angle measurements, performed on coatings with no wax sizing (inset of Figure 5a,c,e,g) and after a second dip-coating with an additional layer of carnauba wax (inset of Figure 5b,d,f,h). Filter paper coated with neat PHB (8.5 *w*/*v* %) without non-solvent were taken as a reference, before and after wax coating, respectively (inset of Figure 5i,j). The contact angle of neat PHB coated paper is only 70° and comparable with values reported in the literature [10]. For the PHB-MP-coated paper at low concentrations, a contact angle of 105° was obtained with stability up to 10 s, as PHB-MP provides only a partial surface coverage (Figure 5a, inset). A higher and more stable contact angle of 122° was obtained for well-developed particles formed at higher concentrations, which almost cover the full paper surface (Figure 5e, inset). After wax sizing the PHB-MP-coated papers, an increase in contact angles is attributed to the hydrophobic action of wax. A reference contact angle value of 112° is the lowest over the neat PHB coating with wax, presenting a flat surface morphology (Figure 5j, inset). The higher contact angles of 129° (Figure 5b, inset) and 144° (Figure 5f, inset) were obtained after deposition of wax over the PHB-MP structured coatings. As such, the highest contact angles are obtained at the highest PHB and non-solvent concentrations, where the individual cellulose fibers are favorably coated with PHB-MP.

#### 2.2.2. Interactions between PHB-MP and the Paper Substrate

**Figure 6:**
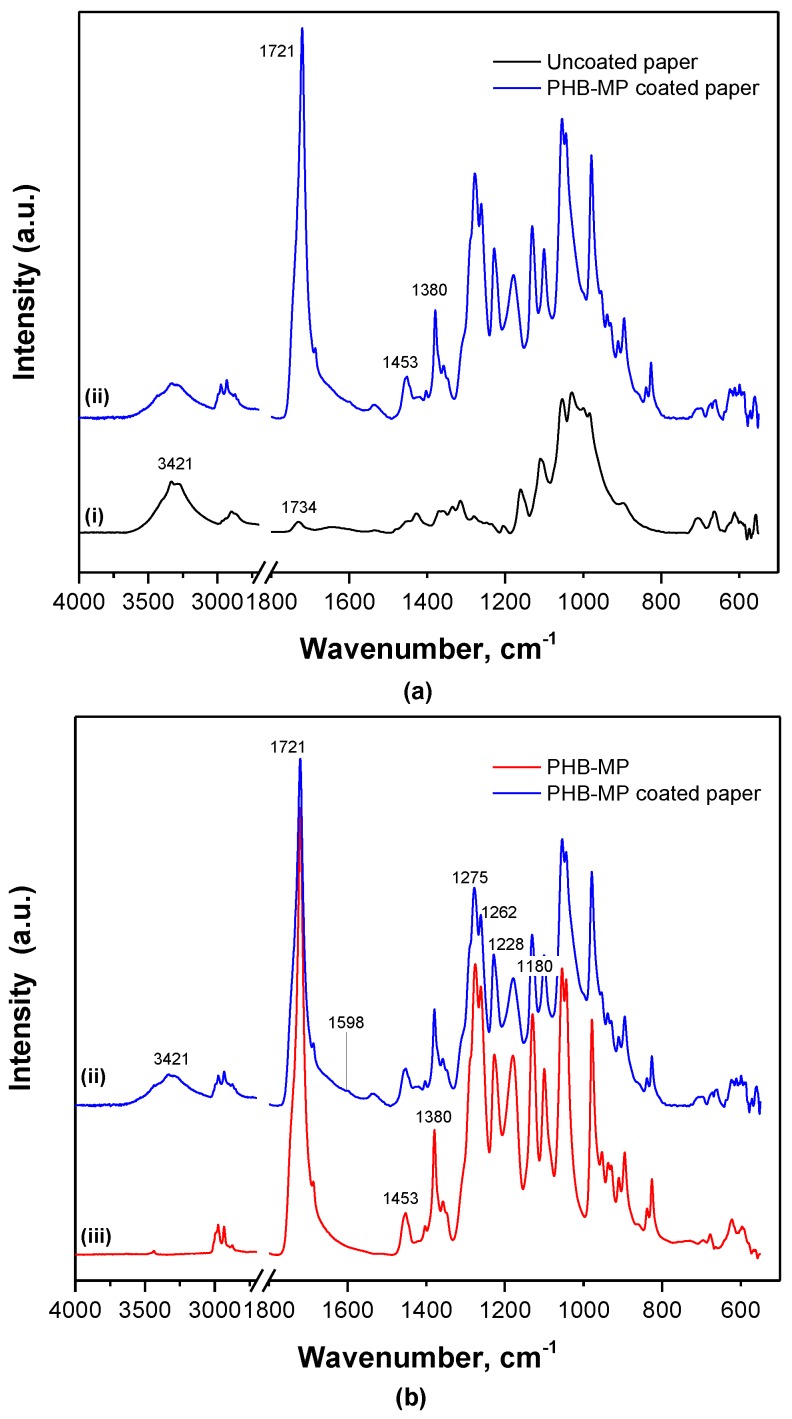
FTIR spectra showing the interactions between PHB-MP and cellulose fibers of the paper substrate: (**a**) spectra normalized over non-overlapped region, 730–645 cm^−1^; (**b**) spectra normalized over the PHB insensitive crystalline band, 1453 cm^−1^, for (i) uncoated filter paper, (ii) PHB-MP coated paper and (iii) PHB-MP powder.

Based on FTIR spectroscopy, some detailed interactions between PHB-MP and cellulose fibers are illustrated in Figure 6. The spectra for uncoated and PHB-MP-coated paper (Figure 6a) were normalized over the absorption region 730–645 cm^−1^ (bands assigned to paper substrate, not overlapping with PHB), referred in the literature to C–H and C–C stretching [33]. The band at 1734 cm^−1^ is referred to the acetyl ester group and uronic hemicellulose and/or ester bonds of the carboxyl groups present in the hemicellulose and lignin [34]. The lower intensity of the 3421 cm^−1^ band (O–H stretching in cellulose) for coated paper compared to uncoated paper suggests the interaction between PHB-MP and cellulose fibers through hydrogen bonding. The spectra for PHB-MP-coated paper and PHB-MP powder (Figure 6b) were normalized over the 1453 cm^−1^ band, which is insensitive for changes in crystallinity [30]. The broadening in the wavenumber region 1500–1800 cm^−1^ and the formation of a very small band at 1598 cm^−1^ for PHB-MP coated on paper compared to PHB-MP powder are observed: these have also been reported earlier in the case of transgenic PHB production in plants and represent intrinsic esteric and hydrogen bonding with the cellulose fibers [28]. Therefore, it seems that the bonding between the PHB-MP and fibers of paper substrate can be related to the esteric and hydrogen bond formation. This confirms the favorable interaction and deposition of PHB-MP near the cellulose fibers, as observed in previous micrographs. In addition, the crystallinity index for PHB-MP powder (*I*_1228_/*I*_1443_ = 4.5) is higher than for PHB-MP-coated paper (*I*_1228_/*I*_1443_ = 3.5): the interaction between PHB-MP and cellulose fibers of paper clearly restricts the crystallization of PHB, due to confinement of the polymer chain mobility of PHB-MP in contact with paper substrates.

### 2.3. Second Approach: Application of PHB-SP as Paper Coating

#### 2.3.1. Morphological Analysis (SEM) and Water Contact Angle Measurements

**Figure 7:**
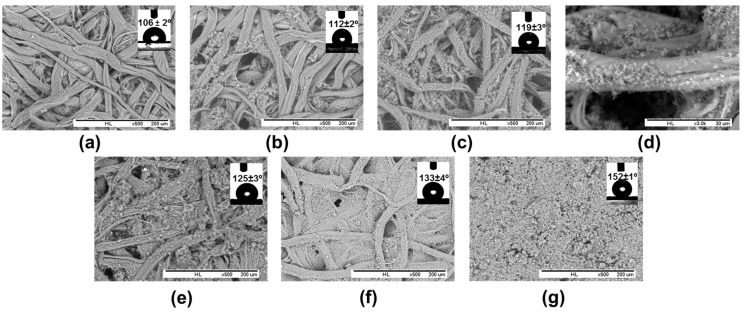
Scanning electron microscopy for PHB-SP- or PHB-SP/NFC-coated papers from suspensions with different concentrations PHB-SP and NFC: (**a**) non-coated reference filter paper, no PHB-SP and no NFC; (**b**) 5 *w*/*v* % PHB-SP, no NFC; (**c**) 20 *w*/*v* % PHB-SP, no NFC; (**d**) detail of 20 *w*/*v* % PHB-SP, no NFC; (**e**) 5 *w*/*v* % PHB-SP, 1 wt % NFC; (**f**) 5 *w*/*v* % PHB-SP, 2 wt % NFC; (**g**) 5 *w*/*v* % PHB-SP, 7 wt % NFC; insets are water contact angles after the application of an additional wax layer over the coated papers.

In the second approach, the PHB-SP were deposited onto paper by dip-coating filter paper samples into the prepared PHB-SP suspension and drying at 103 °C for 30 min. Filter paper without PHB-SP/NFC coating was taken as a reference sample (Figure 7a). Two different particle suspensions with a low concentration (5 *w*/*v* % PHB-SP) and a high concentration (20 *w*/*v* % PHB-SP) were used to evaluate the differences in surface coverage on paper. In contrast with the observations for PHB-MP, however, a fully-covered paper substrate with a continuous coating layer could not be obtained at low PHB-SP concentrations (Figure 7b), nor at high PHB-SP concentrations (Figure 7c). The differences in crystallinity between PHB-MP and PHB-SP may be the reason for reduced interactions between the particles and cellulose fibers (see further Section 2.3.2). Interestingly, at a higher concentration of PHB-SP suspension, slight improvement in PHB-SP retention can be seen over the paper fibers localized only near the microfibrils protruding from the paper fibers (detailed in Figure 7d). With this observation, the idea of using nanofibrillated cellulose (NFC) as a binder in order to enhance the retention for PHB-SP at the paper surface originated.

In order to enhance the retention of PHB-SP at the paper surface, NFC was added from an aqueous stock solution and mixed into a coating formulation with PHB-SP suspension (5 *w*/*v* %). The suspension of PHB-SP/NFC with variable amounts of NFC (1, 2, 5 and 7 wt %) was applied over paper samples by means of the dip-coating method, and the corresponding SEM images of coated paper surfaces are presented in Figure 7. The addition of a small fraction of NFC (1 wt %) resulted in a higher retention of PHB-SP on the paper surface (Figure 7e): notably, small concentrations of NFC already enhanced the retention of the more crystalline PHB-SP through favorable interactions (see further Section 2.3.2). The further addition of NFC (2 wt %) enhanced the retention of PHB-SP in parallel with filling the inter-fiber pores to a greater extent (Figure 7f). Finally, a coating composition with 7 wt % NFC resulted in a full coverage of the paper surface (Figure 7g). The progressive addition of NFC signifies the importance of NFC as a binding agent and allows one to increase the retention of PHB-SP on the paper surface towards the formation of a fully-protective coating layer. The higher concentrations of NFC (above 7 wt %) resulted only in a higher coating thickness without having any other beneficial effect (no further increase in contact angle; see below). Therefore, the addition of 7 wt % NFC in the PHB-SP coating suspension was estimated as an optimum amount required to retain a large amount of particles at the surface and form a fully-covering coating layer, resulting in good functionality.

The surface hydrophobicity of the PHB-SP/NFC coated papers was analyzed by static water contact angles, after the additional dip-coating by plant wax solution (Figure 7, insets). Before wax sizing, however, the hydrophilicity of NFC was predominant for PHB-SP/NFC-coated papers (contact angles < 40°). Even though the hydrophilic NFC network embedding the PHB-SP provides better surface coverage, the presence of NFC masks the hydrophobic character of PHB-SP, and therefore, additional wax coating was required. After wax sizing, the contact angles for a reference paper with no PHB-SP/NFC coating were the lowest at 106° (Figure 7a, inset). Water contact angles further increased with PHB-SP concentration (no NFC) from 1° to 12° (Figure 7b, inset) to 119° (Figure 7c, inset); higher contact angles of 125° (Figure 7e, inset), 133° (Figure 7f, inset) and 152° (Figure 7g, inset) were obtained for PHB-SP/NFC coatings with progressively increasing NFC concentrations. Here, the surface hydrophobicity of the coated papers is largely controlled by the high retention of PHB at the paper surface and wax layer on these structures. Furthermore, the formation of wax crystals was not observed in the magnified SEM images (see Figure S1). Therefore, it can be assumed that the wax layer has been deposited as a uniform film.

#### 2.3.2. Mechanisms of Interactions between PHB-SP/NFC and the Paper Substrate

Considering the higher crystallinity of PHB-SP compared to PHB-MP (see before), the PHB-SP are more inert and resistant to the formation of esteric or hydrogen bonds with cellulose fibers of paper, resulting in reduced adhesive properties (refer to the poor surface coverage). After several washing cycles with water, most of the PHB-SP particles embedded in the network of NFC were removed, as water breaks the NFC-paper fiber inter-hydrogen bonding and thus releases the trapped PHB-SP (see Figure S2). However, after the additional wax sizing, the particles remained on the surface (as in Figure 7) after washing due to hydrophobic protection by the wax. Attempts have also been made to fix the PHB-SP onto the paper prior to the wax coating by performing thermal curing on the coated samples at temperatures of 150 °C and 180 °C for 30 min (see Figure S3). Curing at 150 °C resulted in improvement in the adhesion of PHB-SP on the paper fibers due to the interaction between melted PHB and fibers, but at the same time disturbed the nanoscale surface morphology due to uneven melting of PHB-SP, attributed to the differences in the particle sizes. The curing at the higher temperature of 180 °C resulted in almost even melting of PHB-SP along with better adhesion to paper fibers, but the favorable nanoscale surface morphology was then lost, responsible for the higher hydrophobicity. Therefore, the interactions among the PHB-SP/NFC coating components and the paper substrate can then be mainly explained by two aspects: (1) interaction between PHB-SP and NFC components in the coating suspension; and (2) interaction of suspended PHB-SP and NFC with cellulose fibers of the paper substrate.

**Figure 8:**
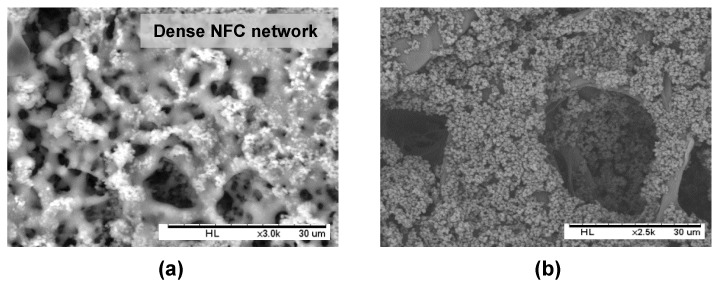
Scanning electron micrographs of filter paper dip-coated with the PHB-SP/NFC suspension having 7 wt % NFC, showing: (**a**) a dense NFC network retaining PHB-SP, when placed under strong X-rays for 5 min; and (**b**) retention of PHB-SP over the single fiber of paper after diluting the coating suspension (added 4 mL distilled water).

The interaction between PHB-SP and NFC in the coating suspension can be understood from the results obtained in the previous sections (Figure 7), where NFC enhanced the retention of PHB-SP at the paper surface, thereby acting like a binding agent. This interaction mechanism lies in the geometrical entrapment of the PHB-SP within the very fine fibrillar network of NFC: similar observations have been reported while using NFC for enhanced retention of precipitated calcium carbonate nanoparticles over the paper [35]. This mechanism can be further evidenced by the observations in Figure 8a, after leaving the coated paper (5 *w*/*v* % PHB, 7 wt % NFC) under a strong electron beam of the SEM for 5 min: the radiation resulted in melting of the PHB-SP and hence clearly revealed the dense NFC fibrillar network covered with PHB-SP.

The interaction of the PHB-SP/NFC coating suspension with the paper fibers can be ideally realized through the chemical interaction between NFC and the paper fibers: the latter is favored because both NFC and cellulose fibers are chemically similar (having surface O–H groups), whereas PHB-SP is rather physically adhered over the paper fibers through entanglement in the NFC network. The very strong attractive forces between NFC and paper fibers occur through hydrogen bonding and van der Waals forces. In addition to the surface chemistry, the high surface area and aspect ratio of NFC further promote the intimate contact between NFC and the paper fibers. The strong interaction of NFC with the paper fibers may assist the good retention of PHB-SP on single paper fibers, as shown in Figure 8b, after diluting the coating suspension (5 *w*/*v* % PHB-SP, 7 wt % NFC) by adding 4 mL of extra distilled water: as such, the PHB-SP deposits on single paper fibers can be realized, and the adhesive properties are mediated by the presence of NFC.

### 2.4. Coating Weight and Coating Thickness

After dip-coating paper samples as detailed in the Experimental Section, the coating weight for a one-side coated paper was calculated accordingly after the first dip-coating (i.e., PHB particles over paper) and second dip-coating (i.e., wax). The net coating weight per unit surface area, net coating thickness and corresponding water contact angles before and after wax coating are summarized in Table 1. The consumption of wax (wax coating weight) applied to the various PHB-coated papers under the same application conditions differs and depends on the coating type. The higher wax coating weight on filter paper (2.5 mg) in comparison with the filter paper coated prior with neat PHB (2.2 mg) indicates the migration of wax inside the paper web in absence of a continuous thick layer of PHB. Otherwise, more wax is consumed for PHB-MP-coated paper (2.8 mg) than PHB-coated paper (2.2 mg), due to the combined effect of wax migration and the presence of particles that increase the total surface area to be covered with wax. In the case of PHB-MP-coated paper with higher concentrations of PHB (see Figure 5e,f), a thicker porous coating layer (thickness = 40 µm) providing almost full surface coverage to the paper surface could retain a higher amount of wax (3.2 mg) inside the porous coating structure. Similarly, for PHB-SP/NFC-coated papers, a gradual increase in wax consumption (2.8–5.5 mg) was measured with increasing net coating thickness (15–50 µm), in parallel with the higher NFC concentrations (0–7 wt %). The latter can most likely also be attributed to an increase in thickness of the porous coating layer and, hence, a higher uptake of wax. In addition, a gradual increase in PHB-SP/NFC coating weight (1–10 mg) with the increase in NFC concentrations (0–7 wt %) can be attributed to the binding effect of NFC for PHB-SP, as discussed previously.

In summary, the water contact angles on different coating types are also included in Table 1, as they were discussed throughout this paper. It is important to note that the paper with neat PHB consumes the maximum amount of coating material as indicated by the highest net coat weight (39.8 g/m^2^) and thickness (65 µm), but provides the lowest contact angles. Otherwise, the coatings with micro- to nano-sized-structured PHB particles reduce the coating weight and the use of raw materials, while providing higher surface hydrophobicity. Therefore, a critical selection of bio-based ingredients in the coating formulation and good control over their presentation at the paper surface may favorably enhance the surface hydrophobicity, which might be an interesting route for future applications as protective coatings for packaging papers.

## 3. Experimental Section

### 3.1. Materials

The neat PHB pellets were purchased from Metabolix GmbH (Köln, Germany) having the trade name of Mirel M2100 with a molecular weight M_w_ = 3.7 × 10^5^ g·mol^−1^ and polydispersity (M_w_/M_n_) in between 1.60 and 1.85. A nanofibrillated cellulose suspension (NFC, E167, 2% consistency) was obtained from VTT Technical Research Centre of Finland (Espoo, Finland). Whatman Grade 4 qualitative filter paper (diameter of 90 mm, thickness 90 µm, grammage of 92 g/m^2^) was used as the paper substrate. The filter paper samples were cut into dimensions of 5 × 1.2 cm^2^ and conditioned at 60 °C for 12 h before use. The carnauba wax was obtained from Basin, N.V. (Wingene, Belgium). Dimethylformamide (DMF, 94%), chloroform (CF, 99%), tetrahydrofuran (THF, 99.9%) and polyvinyl alcohol (PVA, 87%–90% hydrolyzed) were purchased from Sigma Aldrich Chemical Ltd. (München, Germany).

### 3.2. Synthesis of PHB Microparticles and Submicron Particles and Deposition as Paper Coating

The PHB-MP were prepared by dissolving the neat PHB pellets in DMF (8.5 *w*/*v* %) at 150 °C for 10 min under continuous stirring. An amount of 400 µL absolute ethanol and distilled water (9:1) mixture was subsequently charged into 5 mL PHB-DMF solution under continuous stirring for 1 min. The resulting suspension was oven dried at 70 °C for 5 h to facilitate the solvent evaporation, leading to the formation of structured PHB-MP powder.

**Figure 9:**
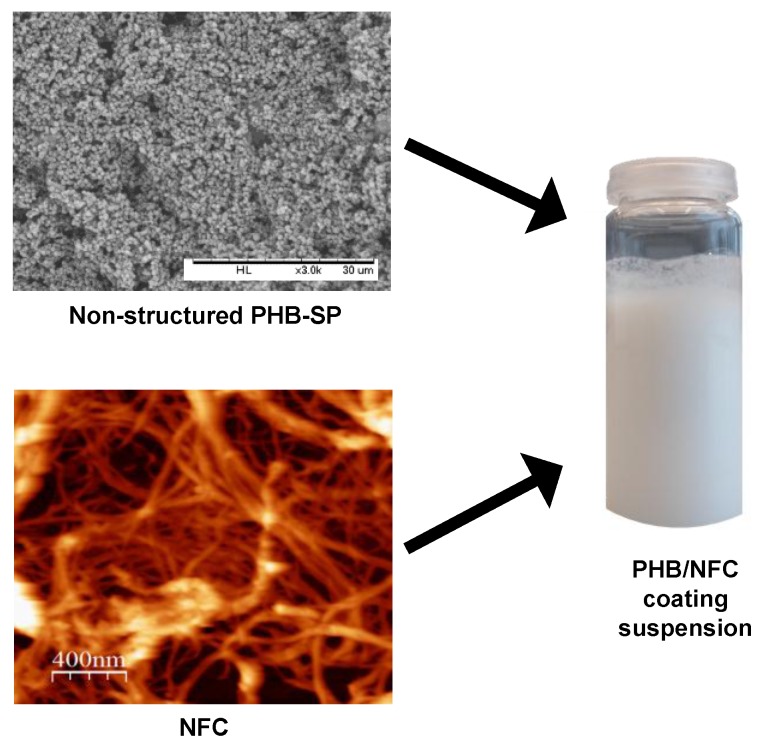
Scheme for preparing PHB-SP/NFC coating slurry used for paper coatings.

The PHB-SP were prepared according to a previously-established oil-in-water (o/w) emulsion solvent evaporation method, with slight modifications [25]. Briefly, the neat PHB pellets were dissolved in chloroform (5 *w*/*v* %) at 70 °C for 15 min and later emulsified in distilled water containing PVA (0.1 *w*/*v* %) for 15 min, using an ultrasonicator UW2200 (Bandelin Electronic, Berlin, Germany). The obtained emulsion was fed into a rotary evaporator (Heidolph, Schwabach, Germany) for solvent evaporation at 200 mbar pressure and 40 °C for 2 h. The PHB-SP were collected by centrifugation at 10,000 rpm for 5 min (F15 6X100YMultifuge, Thermo Scientific, Waltham, MA, USA), washed several times with distilled water to remove PVA and finally stored after freeze-drying (ALPHA 12 LD plus freeze dryer, Martin Christ, Osterode am Harz, Germany). For preparation of the coating formulations, the dried PHB-SP were mixed with an aqueous suspension of nanofibrillated cellulose or NFC (prepared as a 0.4 wt % stock suspension) by ultrasonication in variable concentration ratios (PHB-SP/NFC = 99:1, 98:2, 95:5 and 93:7 wt % on a dry basis) and adjusted accordingly with distilled water to obtain a total of 10 mL of coating slurry, as shown in Figure 9. As such, 500 mg of PHB-SP powder were mixed with variable amounts of NFC (5, 10, 25 and 40 mg on a dry basis) to obtain the mentioned PHB-SP/NFC weight fractions in the coating suspension. As a reference coating formulation and to demonstrate the advantageous effect of NFC, two PHB-SP coating suspensions without NFC were prepared by dispersion of 500 and 2000 mg of PHB-SP in 10 mL of distilled water, corresponding to 5 and 20 *w*/*v* % PHB-SP suspensions, respectively.

For preparing PHB-MP-coated papers, the filter paper samples were dip-coated in the suspension of PHB-DMF/ethanol-water and dried at 70 °C for 5 h. For preparing PHB-SP-coated papers, the filter paper samples were dip-coated into different coating suspensions of either PHB-SP or PHB-SP/NFC and cured at 103 °C for 30 min to evaporate water. Finally, the PHB-MP- and PHB-SP-coated papers were sized by dip-coating in a solution of plant wax in THF solution (6.6 *w*/*v* %) and cured again at 103 °C for 15 min to evaporate the solvent.

### 3.3. Characterization of PHB Microparticles and Submicron Particles and Coated Papers

The morphologies of PHB-MP and PHB-SP in powder form and the corresponding coated paper surfaces were analyzed by scanning electron microscopy (SEM), using a tabletop microscope TM 3000 (Hitachi, Krefeld, Germany). Although the technique allows researchers to work with samples without a sputtered gold film due to the regulation of the vacuum, better results were obtained after deposition of a thin gold layer over the sample. The diameters of the particles were obtained by measuring the diameters of 100 random particles and were reported as an average value with the standard deviation, along with the range of particle diameters.

The thermal properties of PHB-MP and PHB-SP were determined by thermogravimetric analysis or TGA using the Pyris1 equipment (PerkinElmer, Rodgau, Germany) and differential scanning calorimetry or DSC using the DSC 8500 equipment (PerkinElmer, Rodgau, Germany). For TGA, sample weights of 5 mg were heated from 50 to 400 °C at a rate of 10 °C/min in a flowing nitrogen atmosphere. For DSC, each sample weight of 5 mg was heated from −40 to 200 °C at a rate of 10 °C/min, repeated 3 times per sample and reported as average values with the statistical standard deviation.

The chemical composition of PHB-MP, PHB-SP and the paper coating was determined by attenuated total reflection Fourier-transform infrared spectroscopy or ATR-FTIR on a diamond/ZnSe crystal (9 bounces) using a Spectrum 65 equipment (Perkin Elmer, Rodgau, Germany), collecting spectra between 4000 and 550 cm^−1^ wavelengths with a resolution of 4 cm^−1^ and averaged over 32 scans.

The topography of pure NFC pulps at the nanoscale was studied with tapping-mode atomic force microscopy (AFM), using Nanoscope III with a tube scanner from Digital Instruments (Veeco, Santa Barbara, CA, USA) and silicon tips with stiffness *k* = 50 N/m and a resonant frequency of 360 kHz (PPP-NCH, Nanoandmore, Wetzlar, Germany).

The coating weights over the filter paper samples of dimensions of 5 × 1.2 cm^2^ (surface area of 6 cm^2^) were determined by subtracting the coated paper weight by the uncoated weight of filter papers (after conditioned at 60 °C for 12 h). After depositing the PHB-MP or PHB-SP/NFC coating slurry, the final weights of the coated papers were determined only after curing at 103 °C for 30 min to evaporate water. This weight was then subtracted from the weight of uncoated paper to obtain the two-sided coating weight, which was further reduced to half to obtain the one-sided coating weight of the PHB-MP or PHB-SP layer obtained after the first dip-coating step. Similarly, the one-sided coating weights of an additional wax layer applied during a second dip-coating step were determined. Finally, the net coat weight per unit surface area of coated papers was determined by dividing the total coating weight (PHB/NFC + wax, in mg) to 6 cm^2^ and reporting the final values in g/m^2^.

The thickness of the coated papers was measured with a digital caliper at five different places per sample and reported as average values with the statistical standard deviation. The coating thickness was then deduced after subtracting it from the thickness of uncoated paper to have the two-sided coating thickness that was further reduced to half to obtain the one-sided coating thickness over the coated papers.

The hydrophobicity of the coated papers was determined by static contact angles of deionized water measured on the Digidrop equipment (GBX, Romans sur Isere, France) by placing a droplet volume of 4 μL over 60 s and fitting its geometry with a tangent fitting procedure. The measurements were repeated 3 times per sample and reported as average values with the statistical standard deviation.

## 4. Conclusions

Two approaches were developed to fabricate the fully-bio-based paper coatings with enhanced surface hydrophobicity, where polyhydroxybutyrate structured microparticles (PHB-MP) and sub-micron particles (PHB-SP) were first synthesized and deposited over filter papers as coating pigments and later additionally sized by plant wax solution through a facile dip-coating method.

In the first approach, the synthesis of PHB-MP resulted in the formation of internally-structured particles with relatively low crystallinity. Due to the favorable interactions with paper fibers, the PHB-MP were preferentially deposited onto the paper fibers: after drying, interactions between PHB-MP and cellulose fibers were observed through hydrogen bonding and esterification reactions. Depending on the concentrations of PHB and non-solvents, paper coatings with either deposition onto single fibers (low concentrations) or full surface coverage (higher concentrations) can be obtained. An increase was seen in the intrinsic hydrophobicity for PHB-MP-coated papers with static contact angles of 105°–122°. An additional wax coating over PHB-MP-coated paper resulted in a further increase in contact angles from 129° to 144°. As such, the highest contact angles are obtained at the highest PHB and non-solvent concentrations, where the individual cellulose fibers are favorably coated with PHB-MP.

In the second approach, the PHB-SP were synthesized with higher crystallinity, and consequently, lesser affinity with the paper surface was noticed. Therefore, a full surface coverage could not be obtained by increasing the particle concentrations in the coating suspension. As such, the addition of NFC into the coating suspension was crucial in enhancing the retention of the PHB-SP particles at the paper surface, up to a maximum content of 7 wt % NFC, which was required for completely and homogenously covering the paper surface. An additional wax coating over the PHB-SP/NFC-coated paper provides contact angles of 112°–152° with the progressive increase in NFC concentrations. As such, the hydrophobicity and morphology of coatings was highly dependent on NFC as a binding agent, causing entrapment of PHB particles in the fibrillar network and anchoring to the paper fibers.

## Figures and Tables

**Table 1:** One-sided coating weight and thickness for PHB-MP- and PHB-SP/NFC-coated papers, along with the summary of corresponding contact angle measurements.

Coating Type	PHB Coating Weight, mg	Wax Coating Weight, mg	Total Coating Weight, mg	Net Coat Weight, g/m^2^	Net Coating Thickness, µm	Contact Angle before Wax Coating, °	Contact Angle after Wax Coating, °
No coating (filter paper)	-	2.5 ± 0.1	2.5 ± 0.1	4.2 ± 0.2	5 ± 0.5	<40 *	106 ± 2
Neat PHB	21.7 ± 1.2	2.2 ± 0.1	23.9 ± 1.2	39.8 ± 2.0	65 ± 1	70 ± 1	112 ± 1
PHB-MP 1	6.1 ± 0.3	2.8 ± 0.2	8.8 ± 0.4	14.7 ± 0.6	25 ± 2	105 ± 4	129 ± 3
PHB-MP 2	14.2 ± 0.4	3.2 ± 0.3	17.2 ± 0.5	28.7 ± 0.8	40 ± 2	122 ± 2	144 ± 2
PHB-SP/NFC, 0%	1.0 ± 0.1	2.8 ± 0.1	3.8 ± 0.1	6.3 ± 0.2	15 ± 1	<40 *	112 ± 2
PHB-SP/NFC, 1%	3.5 ± 0.3	3.0 ± 0.1	6.5 ± 0.3	10.3 ± 0.5	20 ± 2	<40 *	125 ± 3
PHB-SP/NFC, 2%	5.5 ± 0.2	4.1 ± 0.4	9.5 ± 0.4	15.8 ± 0.7	25 ± 3	<40 *	133 ± 4
PHB-SP/NFC, 5%	8.5 ± 0.5	5.2 ± 0.3	13.5 ± 0.6	22.5 ± 1.0	35 ± 1	<40 *	142 ± 2
PHB-SP/NFC, 7%	10.0 ± 0.6	5.5 ± 0.4	15.5 ± 0.7	25.8 ± 1.2	50 ± 1	<40 *	152 ± 1

Note: Polyhydroxyalkanoate microparticles = PHB-MP; PHB-MP 1 = low concentration of PHB (8.5 *w*/*v* %) and non-solvent (7.4 *v*/*v* %); PHB-MP 2 = high concentration of PHB (17.0 *w*/*v* %) and non-solvent (14.8 *v*/*v* %); Polyhydroxyalkanoate submicron particles = PHB-SP; NFC = Nanofibrillated cellulose; PHB-SP/NFC, 0%–7% = suspension of PHB-SP and NFC with variable amounts of NFC (1, 2, 5 and 7 wt %); * contact angles < 40° are not stable, and water is sucked immediately into the substrate due to hydrophilicity.

## Supplementary Materials

The following are available online at http://www.mdpi.com/2079-4991/7/1/5/s1.

## Abbreviations

The following abbreviations are used in this manuscript:

PHB	Polyhydroxybutyrate
NFC	Nanofibrillated cellulose fibers
PHB-MP	Polyhydroxybutyrate microparticles
PHB-SP	Polyhydroxybutyrate submicron particles
DMF	Dimethylformamide
THF	Tetrahydrofuran
PVA	Polyvinyl alcohol
TGA	Thermogravimetric analysis
DSC	Differential scanning calorimetry
SEM	Scanning electron microscopy
FTIR	Fourier transforms infrared spectroscopy
AFM	Atomic force microscopy
*X_c_*, %	Degree of crystallinity
