# Supplementary material for: Synthesis of Polyhydroxybutyrate Particles with Micro-to-Nanosized Structures and Application as Protective Coating for Packaging Papers
Source: Nanomaterials (Basel). 2016 Dec 30;7(1):5. doi: 10.3390/nano7010005 (PMC5295195; doi:10.3390/nano7010005)
Supplement: Supplementary file 1 [file nanomaterials-07-00005-s001.pdf]

# Supplementary Materials: Synthesis of Polyhydroxybutyrate Particles with Micro-to-Nanosized Structures and Application as Protective Coating for Packaging Papers

**Vibhore Kumar Rastogi and Pieter Samyn**

Figure S1 illustrates the magnified SEM image of PHB-SP-coated (no NFC) paper sized with plant wax solution, where no micro-/nano-scale fractal wax structures are observed.

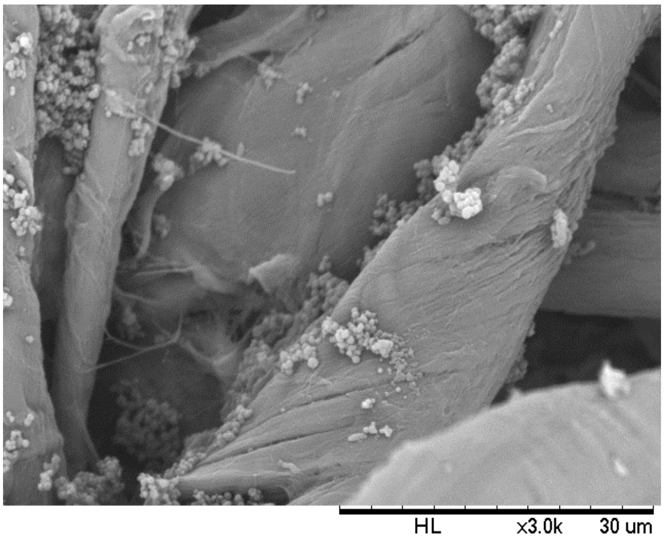


**Figure S1.** Scanning electron micrograph of PHB-SP-coated (no NFC) paper, showing no indications for the presence of micro-/nano-fractal wax structures.

Figure S2 illustrates the magnified SEM image of PHB-SP/NFC-coated (7 wt %) paper (prior to wax coating) that has been washed several times with water and resulted in the removal of most of the PHB-SP particles from the paper surface.

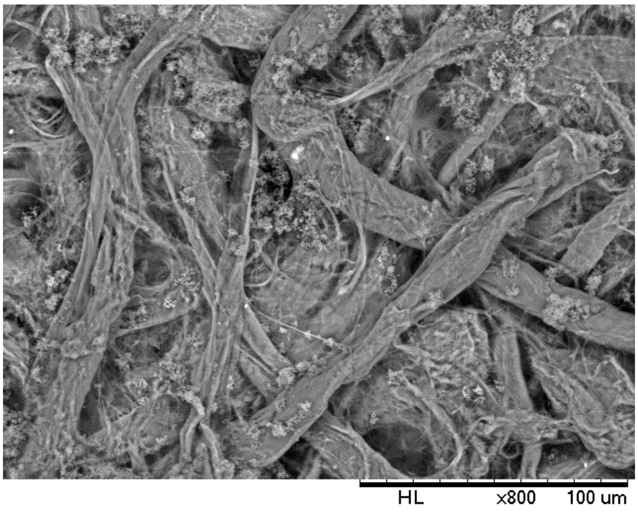


**Figure S2.** Scanning electron micrograph of washed PHB-SP/NFC-coated (7 wt %) paper, showing the retention of few PHB-SP particles after washing.

Figure S3 illustrates the magnified SEM images of PHB-SP/NFC-coated (7 wt %) paper (prior to wax coating) that has been first thermally cured at 150 °C and 180 °C for 30 min and later washed

with water several times. Curing at 150 °C resulted in improvement in the adhesion of PHB-SP directly over the paper fibers due to the interaction between melted PHB and fibers, but at the same time disturbed the nanoscale surface morphology due to uneven melting of PHB-SP, attributed to the differences in the particle sizes. However, curing at higher temperature of 180 °C resulted in almost even melting of PHB-SP along with better adhesion to paper fibers, but the favorable nanoscale surface morphology was then lost, responsible for the higher hydrophobicity.

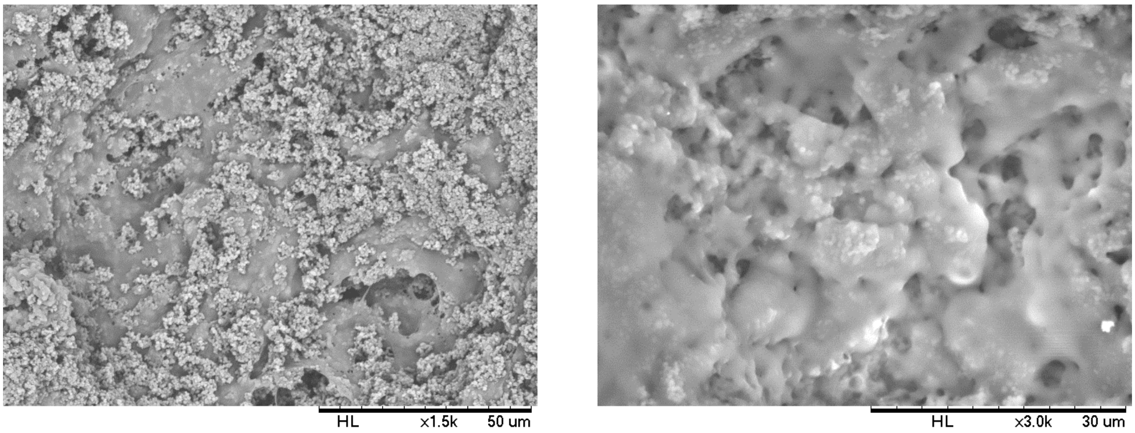


(a) (b)

**Figure S3.** Scanning electron micrograph of washed PHB-SP/NFC-coated (7 wt %) paper thermally cured at (**a**) 150 °C for 30 min and (**b**) 180 °C for 30 min.

© 2016 by the authors. Submitted for possible open access publication under the terms and conditions of the Creative Commons Attribution (CC-BY) license (http://creativecommons.org/licenses/by/4.0/).
